# Multivariate analysis for yield and yield-related traits of sesame (*Sesamum indicum* L.) genotypes

**DOI:** 10.1016/j.heliyon.2020.e05295

**Published:** 2020-10-20

**Authors:** Fiseha Baraki, Zenawi Gebregergis, Yirga Belay, Muez Berhe, Goitom Teame, Mohammed Hassen, Zerabruk Gebremedhin, Assefa Abadi, Weres Negash, Alem Atsbeha, Goitom Araya

**Affiliations:** a Tigray Agricultural Research Institute, Humera Agricultural Research Center, Tigray, Ethiopia; b Ethiopian Institute of Agricultural Research, Wolkite Agricultural Research Center, Addis Ababa, Ethiopia

**Keywords:** Agronomic traits, Grain yield, G×E interaction, Irrigation, Multivariate analysis, Sesame, Agricultural economics, Agricultural soil science, Agricultural technology, Agronomy, Organic farming

## Abstract

Sesame production under irrigation is limited in Ethiopia because of in availability of high yielding varieties, inadequate and inefficient irrigation schemes, and insignificant awareness of producers. This study, comprising 13 sesame genotypes, was conducted around Humera and Werer during 2018 and 2019 under irrigation. The design was randomized completely block design with three replications and the objectives were to develop high yielding genotypes and identify important agronomic traits. Multivariate statistical methods like Additive Main Effect and Multiplicative Interaction (AMMI) model, Principal Component Analysis, Cluster and factor analyses were used. The genotypes (6.22%), environments (42.62) and Genotype × Environment Interactions (25.09%) were statistically (p < 0.001) significant for the agronomic traits. The grain yield in each observation varied from 383 kg/ha to 2044 kg/ha and the grand mean yield was 820.19 kg/ha. The highest mean yield was recorded from G12 (948.6 kg/ha) followed by G4 (938.9 kg/ha) while the lowest was recorded from G8 (703.1 kg/ha). G1, G4, G12, G5, G8, G11 and G13 are identified as unstable genotypes while G2, G3, G6, and G9 are stable genotypes. The genotypes were grouped in to four clusters and cluster-II was characterized as the high yielding cluster and it was also associated with grain yield, pods per plant, branches per plant and thousand seed weight. Branches per plant, pods per plant and thousand seed weight may be most determinant and crucial in developing high yielding sesame varieties. This finding recommends that G4 and G6 are desirable genotypes and can be used for irrigation production.

## 1 Introduction

Sesame (*Sesamum indicum* L*.*), an annual plant that belongs to family Pedelaceae, is the ancient crop that been growing for over 7,500 years in Asia and Africa. Sesame contains about 41.3–62.7% oil (Uzun et al., 2008), 18–25% protein (Borchani et al., 2010), 20–25% carbohydrate (Tunde-Akintunde et al., 2012) and as a result it is grown in many countries as oil seed crop. Furthermore, sesame seeds contain an oil content of 35.6–53.1% in Iran under different levels of irrigation (Kadkhodaie et al., 2014); 47.02 % from white seeds and 49.07% from black seeds in Gizan Saudi Arabia (Bahkali et al., 1998); 45% from white seeds in Korea (Kim et al., 2014); 52.6% from white seeds (Kanu, 2011) and 54.2% from white seeds in Turkey (Ünal and Yalçın, 2008). Generally, oil content highly varies across environments and varieties grown. Sesamin, sesamolin and sesaminol glucosides are the major lignans or antioxidants available in sesame seed, and are curtail for human health (Murata et al., 2017) and sesame is rich in these antioxidants (Hwang et al., 2005). Because of its adaptability even to poor growing conditions it grows in wider areas globally ranging between 25° N and 25° S latitudes (Ashri and Singh, 2007). The world sesame area coverage and production was 9,983,165 ha and 5,531,948 tons respectively with the productivity of 0.55 ton/ha in 2017 (FAOSTAT, 2019). Tanzania, Myanmar, India, Sudan, Nigeria, China, and Ethiopia are the major sesame producers in the world (FAOSTAT, 2019). In addition to rainfed production it is also possible to produce sesame using irrigation and boosting the productivity to 2,000 kg/ha (Uçan et al., 2007) and even up to 2700 kg/ha (Anastasi et al., 2017). However, sesame production under irrigation condition is limited in Ethiopia because of intermingled problems like limited awareness of producers, inefficient irrigation schemes, limited varieties for irrigation production, low commitment of the government and limited research works in developing technologies for irrigation production. Hence, this study aimed to develop new sesame varieties for irrigation production and study the yield and yield components of sesame genotypes under irrigation condition in the study area.

The occurrence of genotype × environment interaction (GEI or G×E interaction) is widely renowned by plant breeders and agronomists. Genotype ranking may different from one environment to other environment since one genotype may be significantly well adapted to a given environment but not for other environment or the ranks of the genotypes may not be changed because of non-significant GEI (Becker and Leon, 1988). If there is change of rank for a given genotype over environments it is called crossovers or qualitative interaction (Gail and Simon, 1985)and such cross over interaction or significant GEI is very important in agricultural production (Lokmal et al., 1995). On the other hand (Baraki et al., 2014), stated that such significant GEI is not completely important rather it is also a constraint in varietal selection. In investigating genotype stability and adaptability there are different statistical methods the methods can be categorized as multivariate and univariate (Delic et al., 2009). The use of multivariate methods is crucial, since the response of G genotype across E environments may be theorized as a pattern in an E-dimensional space (Flores et al., 1998). Genotypes having identical responses can be clustered and their data can be simply summarized using multivariate analysis (Hohls, 1995). To eliminate noise from the data pattern, to summarize the data and to reveal a structure in the data and to get the right results thereby to forward right conclusion and recommendation in a given study from MET it is important to use multivariate analysis (Crossa, 1990).

(Alberts, 2004; Issa, 2009) suggested that Principal component analysis (PCA), Cluster analysis, factor analysis and Additive Main Effect and Multiplicative Interaction (AMMI) are among the commonly used and easy to manipulate and to understand multivariate analyses. The AMMI has been widely used and better than the traditional ANOVA in multi environment trials since it partitions the noise in to further principal components and it provides additional information on a given trait with interesting graphics easy for interpretation (Gauch, 2013). Principal component analysis (PCA) can effectively downsize the structure of a two-way Multi Environment Trial (MET) data matrix of genotypes (G) across different environments (E) dimension in a fewer dimensions and this method is considered to be efficient (Cruz-Castillo et al., 1994). (Crossa, 1990) stated that cluster analysis is a numerical arrangement technique that explains groups of individuals and hierarchical classification, which important to categorizes individuals into groups having the objective of studying associations in the data (Zhang et al., 2008; Baraki et al., 2015a, 2015b; Jinxiong et al., 1995). used cluster analysis and PCA in sesame to group sesame genotypes based on their agronomic traits and the authors suggested to scholars to manipulate PCA to efficiently downsize multiple traits from multiple environments in a fewer and understandable dimension. Furthermore (Daba et al., 2014; Abate et al., 2015; He et al., 2009; Misganaw et al., 2015; Tadesse and Abay, 2011), reported that the use of bi-plots from AMMI model, which is attractive and easy to understand, is crucial to identify stable and high yielding sesame genotypes.

## 2 Material and methods

### 2.1 Description of the study areas

The experiment was conducted in Humera, Tigray region and Werer, Afar region of sesame growing areas in Ethiopia, for two years in 2018 and 2019 under irrigation condition in four locations in a total of eight growing environments. Humera is located in 14^o^15′ N and 36^o^37′ E in an altitude of 632 m above sea level (masl) receiving about 580 mm annual rainfall. The mean minimum and mean maximum annual temperature of the area is 18.8 and 37.6 °C respectively and the soil is characterized as vertisol. Werer is situated at 90^o^16′ N and 40^o^9′ having an altitude of 750 m above sea level (masl) receiving about 590 mm annual rainfall. The mean minimum and mean maximum annual temperature of the area is 26.7 and 40.8 °C respectively and the soil is characterized as vertisol and flui-soil.

### 2.2 Experimental materials and methodologies

25 sesame genotypes were evaluated under irrigation condition in 2017 and 13 relatively high yielding genotypes were selected and promoted to the next breeding stage. As a continuation of this 13 sesame genotypes were used in this trial and the design was randomized completely block design (RCBD) with three replications in all environments. Each plat had 5m and 2m length and width respectively, having 2m and 1m buffer zones between replications and plots respectively with 40 cm and 10 cm spacing between rows and plants respectively. Seeds of the sesame genotypes were sown on well prepared plots and each plot was receiving 25mm of water through farrow irrigation every four days interval until most of the plats in each plot reached to maturity. Furthermore, all other agronomic practices were applied to each plot equally according to the crop and areas recommendations.

### 2.3 Data collected

From the three middle harvestable rows five representative plants were selected randomly and tagged to collect all the under mentioned agro-morphological data for each genotype at each test environments and grain yield was estimated in (kg/ha).

• Days to 50% flowering (DF): The number of days from emergence to which 50% of the population in each plot become flowered.
• Days to 50% maturity (DM): The number of days from emergence to when 50% of the plants in each plot had fully matured.
• Plant height at maturity (PH) (cm): This growth parameter was measured from ten randomly selected and tagged plants from the harvestable rows of each plot with the help of meter tape from ground surface to the top of the plant.
• Length of capsule bearing zone (LCBZ) (cm): A height from the first capsule to tip of the plant, measured using meter tape.
• Number of primary branches per plant (BPP): Branches producing productive capsules will be recorded for randomly selected plants.
• Number of Pods per Plant (PPP): The total number of capsules was counted from ten randomly selected plants at maturity.
• Seeds per pod (SPP): The average number of seeds available in each pods of sesame
• Thousand Seed Weight (TSW): The seed weight (in gram) of 1000 seeds of sesame
• Grain Yield (kg/ha): the total grain yield harvested from the net plot area was weighed using a sensitive balance.

### 2.4 Statistical analysis

Different multivariate statistical methods were used in this study. Combined analysis of variance using the AMMI analysis for grain yield was performed from the mean data of all environments to detect the presence of GEI and Tukey HSD was performed to explain the significant differences among mean grain yields of the genotypes and environments as well. Furthermore, the AMMI1 and AMMI2 bi-plots were sketched and factor analysis was executed using principle components analysis and varimax rotation of provisional factors was done on the data set for changing the geometric space of variables and helps to maximizing loading of variables on particular factor by the help of R-statistical software (Team, 2020) using “metan” package (Olivoto and Lúcio, 2020). Principal Component Analysis (PCA) using PAST (Hammer et al., 2001), Mahalanobis (D^2^) distance (Mahalanobis, 1936) and cluster analysis based on Ward's method (Ward Jr, 1963) using Squared Euclidean distance of the distance metric and standardized variables were performed using Minitab release 16 (Minitab, 2009) to cluster the genotypes based on grain yield and yield related traits. The D^2^ values of the clusters were assumed as the calculated values of Chi-square (χ2) and were tested for significance at 1% probability level against the tabulated values of χ2 for 'P' degrees of freedom, where P is the number of agronomic traits in the study (P = 9) (Urdan, 2016).

## 3 Results and discussion

### 3.1 Genotypes yield performance and variance estimation

The combined sesame grain yield data were subjected to AMMI analysis. This AMMI combine the conventional analysis of variance (ANOVA) with additive and multiplicative parameters in to a single model and it further partitions in to principal components (Gauch, 2013). The results for the sesame agronomic traits (data not shown) and grain yield showed that both the genotypes (G), environments (E) and Genotype × Environment Interactions (G×E interactions) were statistically (p < 0.001) significant indicating the sesame genotypes were significantly different in their genetic potential under irrigation condition and the environments where the genotypes grown were different in different aspects (Tohidi et al., 2020). also reported a significant G, E and G×E interaction in sesame seed yield and oil yield under irrigation conditions. The E, G×E interaction and G effects contributed 42.62, 25.09, and 6.22 % of the total variation respectively. The G×E Interaction was further divided in to a total of seven Principal Component Analysis (PCAs) contributing PCA1 and PC2 about 60.7 and 28.4% of the G×E Interaction respectively (Table 1). There was statistically significant difference in mean grain yield among the sesame genotypes and the highest yield was recorded from G12 (948.6 kg/ha), G4 (938.9 kg/ha) and G13 (907.2 kg/ha) and the lowest grain yield was recorded from G8 (703.1 kg/ha) with grand mean yield of 820.19 kg/ha. Five sesame genotypes viz. G4, G5, G6, G12 and G13 yielded above the grand mean yield while the remaining eight genotypes produced below the average yield. The grain yield of the 13 sesame genotypes across the growing environments showed a statistical significant variation among the environments (Table 2) and the highest (1325.4 kg/ha) and the lowest grain yield (632.2 kg/ha) were recorded from E6 and E2 correspondingly. This indicates sesame grain yield and yield related traits were highly affected by the growing environments. And this is true for sesame both in rain fed (Baraki et al., 2015a; Tantawy et al., 2007; Nath et al., 2001) and irrigation production (El Mahdi et al., 2007; Nadeem et al., 2015; Ebrahimian et al., 2019). (El Mahdi et al., 2007) also reported that a sowing date difference in sesame significantly affects sesame seed yield and yield related traits under irrigation condition and this may be mainly sesame is highly sensitive to temperature. A temperature less than 18 °C can have a negative effect during germination and heat-wave periods above 40 °C inhibits pollination and the formation of capsules (Naturland, 2000; Hansen, 2011). Generally, sesame is very sensitive to different biotic and abiotic stresses and the occurrence of any of the biotic and abiotic stresses can significantly affect both the quality and quantity of sesame. Furthermore, inappropriate management practices and the use of conventional technologies like low yielding varieties and poor agronomic and management practices can significantly affect the sesame yield and quality.

**Table 1:** Combined AMMI ANOVA for grain yield of the sesame genotypes.

Source	DF	SS	MS	% SS	% GEI	Accumulation % GEI
ENV	7	13260972	1894425∗∗∗	42.62	.	.
REP (ENV)	16	529721	33108	1.70	.	.
GEN	12	1935831	161319∗∗∗	6.22	.	.
ENV∗GEN	84	7806326	92932∗∗∗	25.09	.	.
PC1	18	4602919	255718∗∗∗	14.79	60.7	60.7
PC2	16	2155338	134709∗∗∗	6.93	28.4	89.2
PC3	14	347091	24792^**ns**^	1.12	4.6	93.7
PC4	12	242588	20216 ^**ns**^	0.78	3.2	96.9
PC5	10	120238	12024 ^**ns**^	0.39	1.6	98.5
PC6	8	90617	11327 ^**ns**^	0.29	1.2	99.7
PC7	6	20808	3468 ^**ns**^	0.07	0.3	100
Residuals	192	8397934	43739		.	.
Total	311	31930785	102671		.	.

**DF**: Degrees of freedom; SS: Sum of squares; **MS**: Mean square; **ENV**: Environment; **Rep**: Replication; **ENV∗GEN**: Environment genotype interaction; **PC**: Principal component; ∗∗∗: significant at p < 0.001; **ns**: non-significant.

**Table 2:** Genotypes mean Grain Yield (kg/ha) across environments.

Genotype Name	Location	Dansha	Bereket	Humera	Dansha	Bereket	Humera	Werer	Werer	Genotype Mean
	Year	2018	2018	2018	2019	2019	2019	2018	2019	
	Gen code	E1	E2	E3	E4	E5	E6	E7	E8	
Hir hir kibebew early set-1	G1	441.3	621	392.3	949.7	813.7	1356.3	721.7	781	759.6^**cd**^
Tejareb kokit sel-3	G2	816	616.3	564.3	854.7	563.7	1121.7	688.7	730.7	744.5^**cd**^
UCR-82-14(N) 209	G3	812	651.7	689.3	960	693.7	1203.3	654	677.3	792.7^**bcd**^
WARC -60 (Different)	G4	1021	691	829.7	919	727	1987.7	683.3	652.3	938.9^**a**^
GojamAzene Yohannes sel-1	G5	994.7	600.3	843	693.3	749.7	1706.7	626	601.3	851.9^**abc**^
Setit-1 (Standard cheek)	G6	1003.3	673.7	852.7	940	770.3	1281.7	690.7	930.3	892.8^**ab**^
NN-027	G7	618	691	573.7	1135.3	740.3	1362.3	631	578.3	791.2^**bcd**^
Hir hir filweha large seeded	G8	581.7	580	493	925.7	721.7	878.3	741.7	702.7	703.1^**cd**^
Tejareb girar	G9	956.7	682	693	809	840.3	1275	609.7	644.3	813.8^**bcd**^
Acc -WW-001 (7)	G10	824.3	591	790.7	711.7	810.3	894	701	757.7	760.1^**cd**^
Hir hir (local cheek)	G11	913.3	572.3	907.7	803	632	750.8	755	730.7	758.1^**cd**^
Hir hir kibebew Airless sel-1	G12	1050.3	624.3	854.7	1043.3	687	1965.7	661.3	702	948.6^**a**^
Maru sel-1	G13	1217.3	623.7	997	872.7	833.7	1447.3	580.7	685.3	907.2^**ab**^
Environment Mean (kg/ha)		865.4^**b**^	632.2^**d**^	729.3^**c**^	893.6^**b**^	737.2^**c**^	1325.4^**a**^	672.7^**cd**^	705.7^**cd**^	
Grand Mean (kg/ha)										820.19
LSD (<0.05)										117.76
CV (%)										25.22

Means followed by same letter are significantly not different.

Hence, side to side genotype evaluation it is important to study the optimum sowing period for sesame under irrigation condition. A box plot is also named as a schematic plot or box-and-whiskers plot is crucial to rapidly summarize and interpret tabular data (Williamson et al., 1989). It displays five descriptive statistics viz. the first and third quartiles, median, and the non-outlying minimum and maximum observations (Spriestersbach et al., 2009; Sun and Genton, 2011).

**Figure 1:**
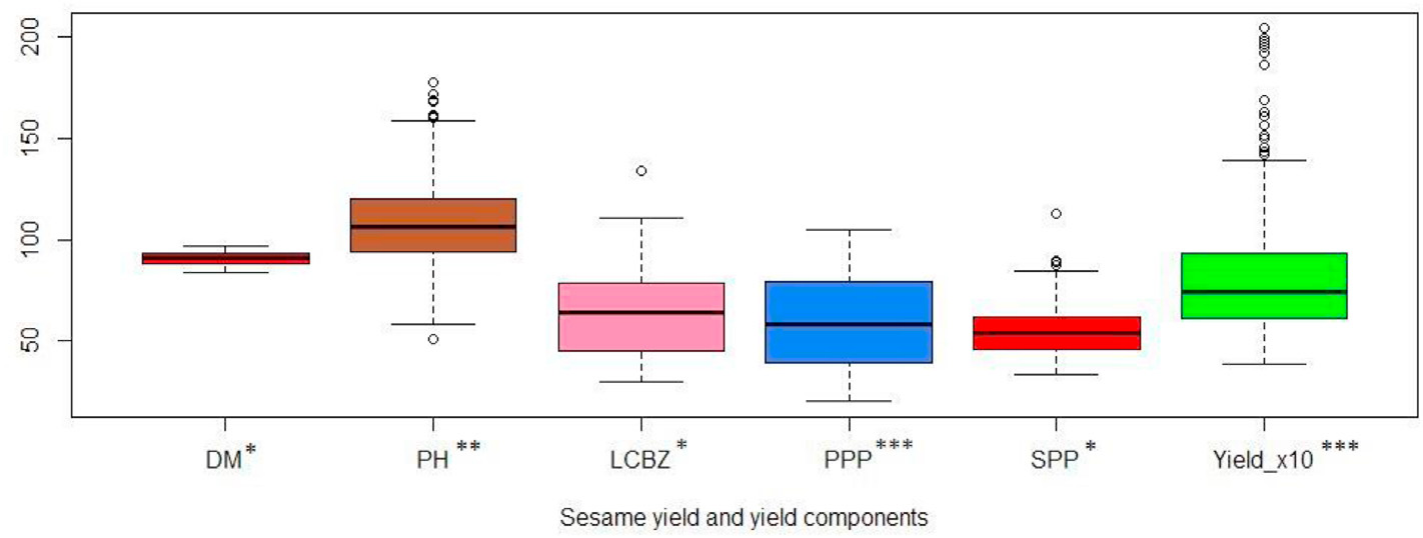
Box plot for agronomic traits of 13 sesame genotypes from 312 observations. DM: Days to 50% maturity; PH: Plant height at maturity (cm); LCBZ: Length of capsule bearing zone (cm); PPP: Number of Pods per Plant; SPP: Seeds per pod; Yield_10: Grain Yield (kg/ha) X10^1^: ∗, ∗∗ and ∗∗∗: Significant at P < 0.05, 0.005 and 0.001 respectively.

A box plot is depicted in (Figure 1) to easily visualize the summary on agronomic traits of the sesame genotypes from each observation (N = 312). The median for grain yield is 744 kg/ha and the first and third quartiles are 610 and 927.8 kg/ha correspondingly. The grain yield in each observation varied from 383 kg/ha to 2044 kg/ha indicating the potential under irrigation condition in these areas is unexploited yet. This result is in accordance with (El Mahdi et al., 2007; Golestani and Pakniyat, 2015; Uçan et al., 2007) who reported a grain yield of 2000 and 1128.92 kg/ha, productivity from irrigation respectively. Irrigation scheme and frequency also highly affects oil content and composition (Kadkhodaie et al., 2014) which needs further study in the study areas. Sesame seed yield is also very low even under rainfed condition in Ethiopia as reported by (Baraki and Berhe, 2019) 650 kg/ha (Daba et al., 2014); 645–881 kg/ha (Gebregergis et al., 2018); 651 kg/ha and (Mekonnen and Mohammed, 2009) 559.2 kg/ha and this is associated to erratic rainfall, low yielding varieties, diseases and insect pest infestation and poor management. This indicates the productivity of all the sesame genotypes used in this study are superior to many other varieties under different managements in Ethiopia.

### 3.2 AMMI model analysis

**Figure 2:**
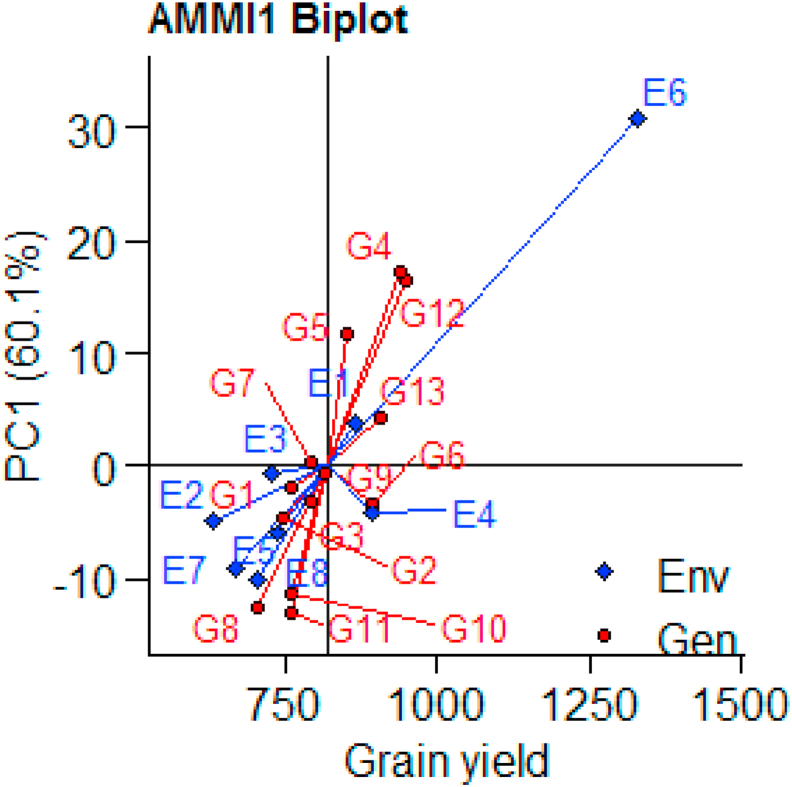
AMMI1 bi-plot to visualize the Yield performance of Genotypes and Environmental (the environment and genotype codes are described in Table 2).

**Figure 3:**
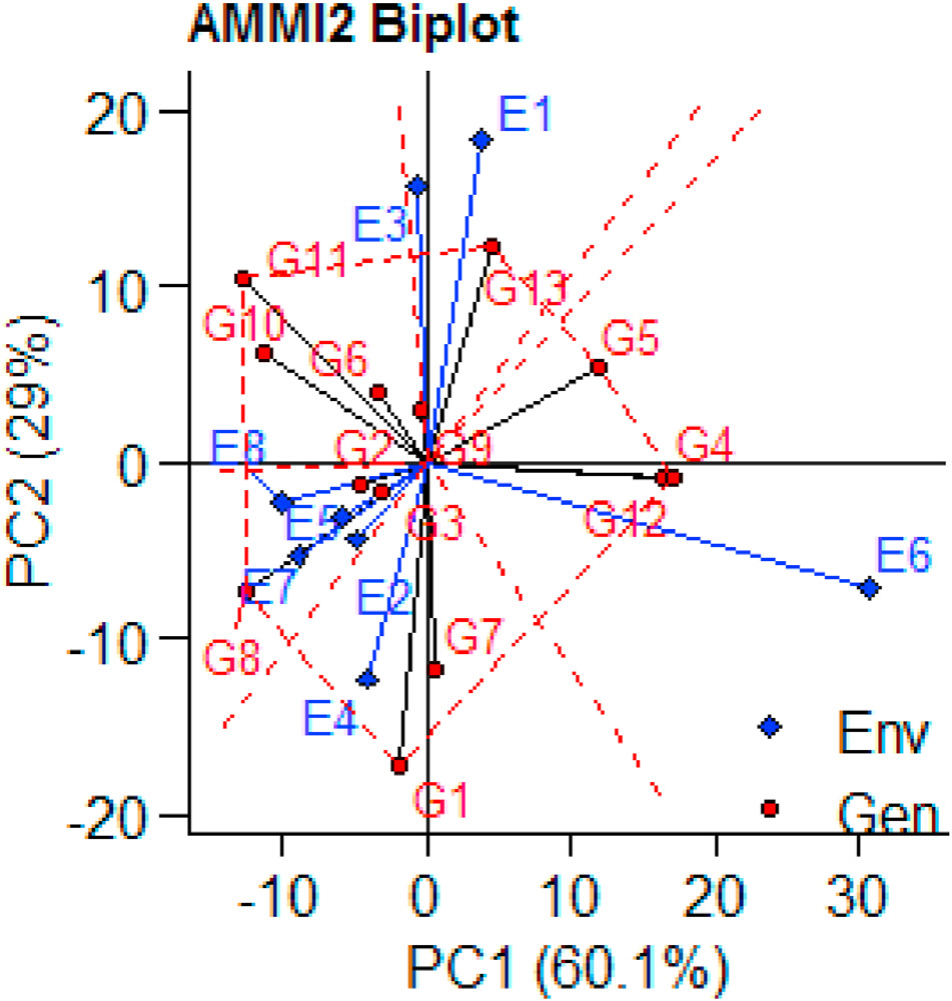
AMMI2 bi-plot to visualize the contribution of the genotypes to the G×E interaction (Baraki et al., 2014; Misganaw et al., 2015; Tadesse and Abay, 2011) also used AMMI1 and AMMI2 bi-plots to identify and recommend high yielding and stable sesame genotypes.

G × E interaction and recapitulating patterns and associations of genotypes and environments is also another importance of AMMI model (Zobel et al., 1988). Furthermore, AMMI model enables breeders to easily visualize and identify high yielding and stable genotypes in attractive and simple graphics. The AMMI1 containing PC1 versus main effects and AMMI2 containing PC1 versus PC2 are generally informative (Gauch and Zobel, 1996). In the AMMI1bi-plot the abscissa represents the mean of the environments and genotypes and the ordinate represents the PC1. Both the genotypes and environments in the right side of the abscissa are high yielding genotypes and environments (above the mean yield) while those in the left side are low yielding genotypes and the environments are unfavorable environments (Yan, 2001; Yan and Rajcan, 2002; Yan and Tinker, 2006). The AMMI1 bi-plot containing average mean of the environments and genotypes in the abscissa and PC1 (60.1%) in the ordinate is depicted in (Figure 2). Consequently, genotypes G4, G5, G6, G12 and G13 which are in the right side of the abscissa produced a grain yield of above mean yield while all the other genotypes produced below mean yield and it is similar to (Table 2). Likewise, environments E1, E4 and E6 in the right side of the abscissa are favorable environments yielding above the average and the other environments in the left side are unfavorable and yielding below the average mean yield. The AMMI2 bi-plot showing PC1 (60.1%) in the abscissa and PC2 (29%) in the ordinate is exhibited in (Figure 3). (Purchase, 1997) pointed out that genotypes with a longest vector length from the origin are unstable with highest contribution for the G×E interaction and hence, are unstable while interaction are stable genotypes. Accordingly, G1, G4, G12, G5, G8, G11 and G13 are the genotypes in the vertexes confirming these genotypes are most unstable ones and G2, G3, G6, and G9 are genotypes near to the origin and are stable genotypes. Hence, both AMMI1 and genotypes with shorter vector length from the origin have minimum contribution for the G×E AMMI2 identified G6 as high yielding and most stable genotype.

### 3.3 Cluster analysis and genetic divergence

**Figure 4:**
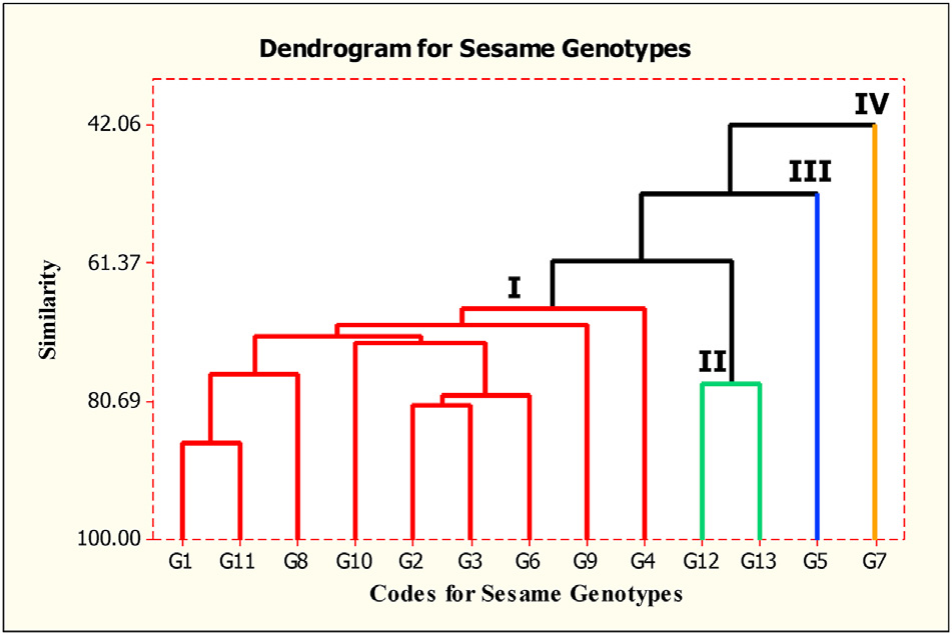
Dendrogram showing the cluster of the sesame genotypes.

Dendrogram was sketched from cluster analysis of the 13 sesame genotypes based on the 9 agronomic traits (Figure 4). Based on the cluster analysis the genotypes grouped in four clusters where cluster I comprised nine genotypes viz. G1, G2, G3, G4, G6, G8, G9, G10 and G11 while cluster-II comprised two genotypes viz. G12 and G13. The third and fourth clusters contained only one genotype each, G5 and G7 genotypes respectively. The mean of the agronomic traits on cluster based is summarized in (Table 3). These results are in accordance to the findings of (Tripathi et al., 2013; Ahmed et al., 2008; Kang et al., 2006). Genotypes in cluster-I are characterized as relatively early flowering genotypes (35.5 days) although they are low yielding (795.82 kg/ha) with smaller thousand seed weigh (2.74 g) than any other cluster. Most importantly, the genotypes in cluster-II had relatively higher mean grain yield (927.76 kg/ha), higher number of branches per plant (3.81) and highest number of pods per plant (61.84). The genotypes in cluster-II also rested on the right side of the AMMI1 bi-plot and are among the high yielding genotypes. High seed yield together with oil content are the most important goals in sesame breeding and in sesame production too. Hence, through using different sesame breeding strategies these genotypes may be very important for developing high yielding varieties in further sesame breeding.

**Table 3:** Mean of the agronomic traits of sesame genotypes by cluster.

Cluster	DF	DM	BPP	PH	LCBZ	PPP	SPP	TSW	Yield
Cluster-I	35.53	90.97	3.59	107.21	63.55	60.23	55.69	2.74	795.82
Cluster-II	36.34	89.51	3.81	104.95	65.05	61.84	51.72	2.81	927.76
Cluster-III	36.32	90.45	3.37	114.70	63.28	56.68	58.28	2.82	851.90
Cluster-IV	36.40	87.61	3.78	105.23	59.13	56.11	50.84	2.86	791.09

G7, the single genotype in cluster-IV is the low yielding (791.09 kg/ha) genotype. However, this genotype is early maturing (87.61 days) and had relatively higher thousand seed weight (2.86 g). Furthermore, this genotype is moderately stable genotype (Figure 3) and hence it is important to use for developing early maturing varieties in drought prone areas which is important to have such early maturing sesame germplasms in the sesame growing areas of Northern Ethiopia. This is because the sesame growing areas in Ethiopia are characterized by erratic rainfall and this is the major reason for lower productivity intermingled with the other constraints. The genotype in cluster-III (G5) is the genotype with no unique characteristics. Generally, genotypes grouped in clusters-I, clusters-II and clusters-IV are with some unique characteristics as described in (Table 3) and hence, these genotypes should be maintained and can be important genetic materials for further sesame breeding. To assess the genetic divergence of the sesame genotypes Mahalanobis (D^2^) distance (Mahalanobis, 1936) was executed and presented in (Table 4). (Urdan, 2016) suggested that the distance values between clusters computed from these pairs of clusters assuming as the calculated values of Chi-square (χ2 = 27.88) were compared for significance at (P < 0.001) against the tabulated values of χ2 for 'P' degree of freedom, where P is the number of agronomic traits of the genotypes considered (9 traits in this study). Accordingly, genetic divergence among all clusters was statistically (p < 0.001) significant indicating that the clusters are highly diverged (Hika et al., 2015; Ganesan et al., 2010; Rahim et al., 2010; Rahman and Munsur, 2009; Reddy et al., 2012). also reported a significant genetic divergence among clusters. The maximum inter cluster distance (423.076) was between cluster-I and cluster-II followed by 184.468 the divergence between cluster-II and cluster-IV (Islam et al., 2018; Sharma and Devi, 2013). suggested that crossing between such genotypes having wider genetic distance could help in developing better heterosis for boosting yield and improve yield related traits in different crops improvement. In such cases, crossing of genotypes in cluster-IV, genotypes characterized with early maturing, with genotypes in cluster-II, genotypes characterized with high yielding, can be fruitful in producing early maturing and high yielding sesame varieties.

**Table 4:** Mahalanobis distance (D^2^) among the clusters.

	Cluster-I	Cluster-II	Cluster-III
Cluster-II	423.076∗∗∗		
Cluster-III	135.64∗∗∗	129.872∗∗∗	
Cluster-IV	60.534∗∗∗	184.468∗∗∗	58.643∗∗∗

∗∗∗ Significant at P < 0.001 (X^2^) = 27.88).

### 3.4 Principal component analysis (PCA)

**Figure 5:**
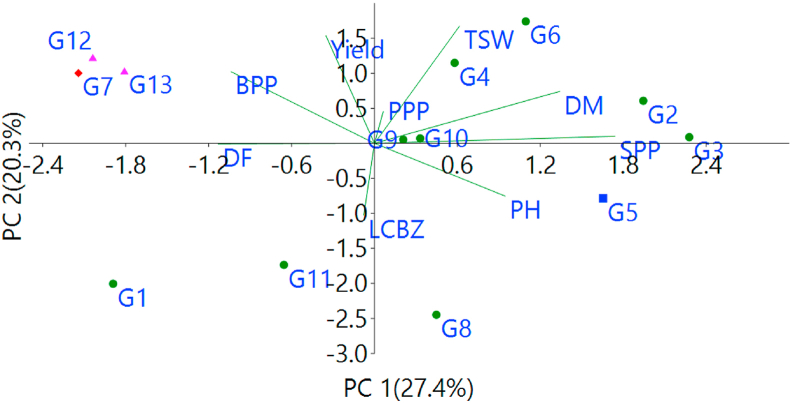
Principal component analysis of sesame genotypes and their traits (the codes for genotypes and traits are as described in Tables 2 and 3 respectively).

**Figure 6:**
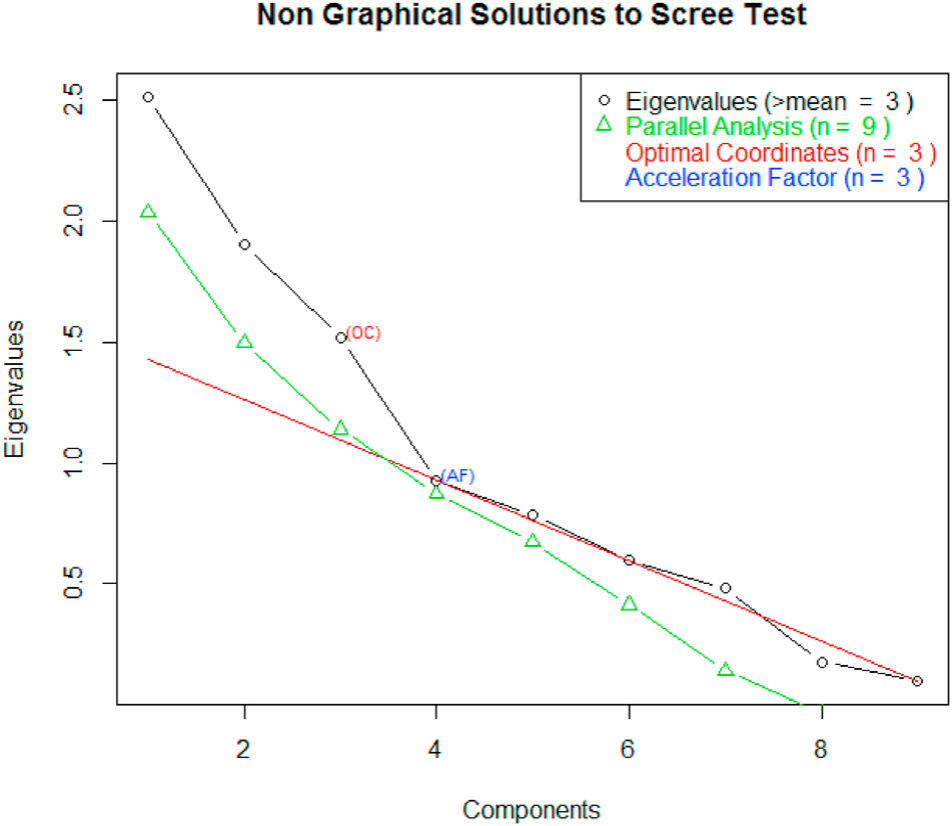
Scree plot for the Principal components of the nine agronomic traits.

Principal component analysis (PCA) is one of the oldest and most popular multivariate techniques. PCA which is classified as non-parametric statistics (Chang and Keinan, 2014) summarizes a data set of observations described by several dependent variables, which are inter-related and its objective is to extract the desired information from the data table (Legendre and Legendre, 1998). An ordination of the genotypes and the agronomic traits, with PC1 (27.4 %) in the abscissa and PC2 (20.3%) in the ordinate, is depicted in (Figure 5) to assess the association of the genotypes and the agronomic traits (Seiler and Stafford, 1985). pointed out that genotypes and variables near to each other are associated to each other while those in opposite direction are negatively associated to each other. Accordingly, G5, the genotype in cluster-III (Figure 4) is positively associated with plant height and seeds per pod while G12 and G13, genotypes in cluster-II, are associated with grain yield, PPP, BPP and TSW indicating these traits may be important to develop high yielding sesame production. G7 is associated with days to flowering while most of the genotypes in cluster-I and cluster-IV are in the opposite direction with grain yield indicating they are low yielding genotypes and this is harmonized with the findings of (Azeez and Morakinyo, 2011; Hika et al., 2015; Menzir, 2012; Baraki et al., 2015b). Furthermore (Furat and Uzun, 2010; Azeez and Morakinyo, 2011; Choi et al., 2017), also used the PCA for sketching bi-plots and to assess the association of genotypes and their agronomic traits.

(Breseghello and Sorrells, 2006) defined factor analysis as a method which identifies the association between and among agronomic traits of a given crop to bind them into underlying factor. It was executed based on principal component analysis and provisional factors were rotated by Varimax method. Using PCA, a scree plot was sketched (Figure 6) to determine the optimum number of factors which can explain most of the variability in the data set. The scree plot showed that a total of nine components were extracted with the eigen values of 2.46, 1.82 and 1.54 in the first three PCs respectively. Accordingly, the optimal coordinate point rested next to the third component indicating that the three components or factors were optimum to explain the variability in the data set. Factor analysis and loadings of the nine agronomic traits is explained in (Table 5). Furthermore, in analyzing the factor analysis the chi square statistic is 4.56 on 12 degrees of freedom and the p-value is 0.971 and hence, test of the hypothesis suggests that 3 factors are sufficient. The first, second and third factors explained 25, 16.4 and 15.2% of variances respectively accounted a total of 56.6% of the factorial alteration. The first and the second factor could explain seven variables each while the third factor explained only six variables. DM and SPP are highly and positively associated with the first factor while TSW and grain yield are highly associated with the second factor indicating that indirect selection of TSW could improve sesame productivity. In the third factor the SPP had high and positive coefficient. On the other hand BPP and LCBZ had negative and high factorial coefficients in the first and second factors respectively (Biabani and Pakniyat, 2008; Asghari et al., 2014; Mansouri et al., 2014). used factor analysis to downsize sesame observations into few number of factors and easy for interpretation and selection of desirable traits which is crucial in plant breeding.

**Table 5:** Factor analysis and loadings of the nine agronomic traits of the 13 sesame genotypes.

Variables	Factor1	Factor2	Factor3
DF	-0.437		
DM	0.623	0.22	0.156
BPP	-0.616	0.333	0.256
PH	0.473	-0.21	-0.11
LCBZ		-0.622	0.486
PPP			0.997
SPP	0.978	0.105	0.165
TSW	0.243	0.83	
Yield	-0.217	0.528	
Loadings	2.247	1.473	1.372
Proportion Variance	0.25	0.164	0.152
Cumulative Variance	0.25	0.414	0.566

## 4 Conclusion

Generally, there was statistically significant G×E interaction in the grain yield of sesame indicating there could be also performance variation across environments and hence, it is important to undertake further studies in identifying optimum planting time and other climatic effects for irrigation production. From this study there are indications that sesame productivity under irrigation condition can be extended up to 2044 kg/ha although the combined mean showed below this (820.2 kg/ha). The AMMI bi-plots declared that G6, the previously released variety for rainfed production, is among the most stable and high yielding genotype. Furthermore, G4, the recently released variety (Setit-2), is also identified as one of the high yielding genotypes and hence, genotypes G6 and G4 can be also recommended for irrigation production, and G12 and G13 with relatively good agronomic traits should be preserved for further breeding program.

Finally, this study suggests that branches per plant, pods per plant and thousand seed weight may be most determinant and crucial agronomic traits in developing high yielding sesame varieties. Hence, sesame breeders and agronomists should pay a due focus on these parameters to boost sesame productivity and quality through exhaustive works on these traits.

## Declarations

### Author contribution statement

Fiseha Baraki: Conceived and designed the experiments; Performed the experiments; Analyzed and interpreted the data; Wrote the paper.

Yirga Belay, Zenawi Gebregergis: Conceived and designed the experiments; Performed the experiments; Wrote the paper.

Muez Berhe, Goitom Teame: Conceived and designed the experiments; Performed the experiments; Wrote the paper.

Mohammed Hassen, Zerabruk Gebremedhin, Assefa Abadi, Weres Negash, Alem Atsbeha, Goitom Araya:Performed the experiments.

### Funding statement

This work was supported by Tigray Agricultural Research Institute.

### Competing interest statement

The authors declare no conflict of interest.

### Additional information

No additional information is available for this paper.
